# Pioglitazone use in Australia and the United Kingdom following drug safety advisories on bladder cancer risk: An interrupted time series study

**DOI:** 10.1002/pds.5508

**Published:** 2022-07-27

**Authors:** Anna Kemp‐Casey, Barbara Mintzes, Richard L. Morrow, Colin R. Dormuth, Patrick C. Souverein, Elizabeth E. Roughead

**Affiliations:** ^1^ School of Pharmacy and Medical Sciences University of South Australia Adelaide Australia; ^2^ Charles Perkins Centre and School of Pharmacy The University of Sydney Sydney Australia; ^3^ Department of Anesthesiology, Pharmacology & Therapeutics University of British Columbia Victoria Canada; ^4^ Division of Pharmacoepidemiology and Clinical Pharmacology Universiteit Utrecht Utrecht The Netherlands

**Keywords:** adverse drug reaction reporting systems events, diabetes mellitus, patient safety, pharmacovigilance

## Abstract

**Purpose:**

National regulators in Australia and the United Kingdom issued safety advisories on the association between pioglitazone use and bladder cancer in July 2011. The Australian advisory noted that males were at higher risk of bladder cancer than females, while the UK advisory highlighted a new recommendation, suggest careful consideration in the elderly due to increasing risk with age. This study examined whether these differences in the advisories had different age‐ and sex‐based impacts in each country.

**Methods:**

Interrupted time series analysis was used to compare pioglitazone use (prescriptions/100000 population) in Australia and the United Kingdom for the 24 months before and 11 months after the July 2011 safety advisories (study period July 2009–June 2012). Separate models were used to compare use by sex and age group (≥65 years vs. <65 years) in each country.

**Results:**

Pioglitazone use fell in Australia (17%) and the United Kingdom (24%) following the safety advisories. Use of pioglitazone fell more for males (18%) than females (16%) in Australia, and more for females (25%) than males (23%) in the United Kingdom; however, neither difference was statistically significant (Australia *p* = 0.445, United Kingdom *p* = 0.462). Pioglitazone use fell to a similar extent among older people than younger people in the United Kingdom (23% vs. 26%, *p* = 0.354), and did not differ between age groups in Australia (both 18%, *p* = 0.772).

**Conclusions:**

The results indicate that differences in the Australian and UK safety advisories resulted in substantial reductions in pioglitazone use at the population level in both countries, however, differences by sub‐groups were not observed.

**Key Points**

- In July 2011, regulators in Australia and the United Kingdom issued safety advisories about the risk of bladder cancer in people using pioglitazone. The UK advisory included new recommendations cautioning he risk for the elderly. The Australian advisory did not contain age based recommendations, but did highlight bladder cancer was more common in men.
- Following the July 2011 safety advisories, use of pioglitazone fell in Australia (17%) and the United Kingdom (24%), however; no sex‐ or aged‐based differences were observed in either county.
- The safety advisories appear to have been effective in reducing pioglitazone use in Australia and the United Kingdom but did not selectively influence use in subgroups.

**Plain Language Summary**

In July 2011, regulators in Australia and the United Kingdom issued safety warnings about the diabetes medicine, pioglitazone, as it is associated with increased risk of bladder cancer. Australia's safety warning noted there was increased risk of bladder cancer in males compared to females, while the UK advisory recommended careful consideration in older patients noting the risk of bladder cancer increasing with age within its new recommendations section. The aim of this study was to determine whether the advisories influenced medicine use, and whether there was any difference in sub‐groups. We compared the use of pioglitazone in Australia and the United Kingdom for 2 years before the safety warning and the 11 months after, focussing on the impact for males versus females, and younger (under 65 years) versus older people (65 years and over). We found that pioglitazone use fell in Australia (17%) and the United Kingdom (24%) following the safety warnings. However, the falls in use were not different for males and females in Australia or between younger and older people in the United Kingdom. The results suggest that the general message communicating pioglitazone and bladder cancer risk was effective in decreasing use of pioglitazone in both countries, but did not result in changes in sub‐groups by age or gender.

## 1 INTRODUCTION

Drug safety advisories were issued in a number of countries in 2011, warning of the potential association between pioglitazone and bladder cancer.^1^, ^2^, ^3^, ^4^, ^5^ Advisories were issued in Australia and the United Kingdom in July 2011, with each emphasizing different background patient risk factors for bladder cancer.^4^, ^5^ The Australian safety advisory stated ‘The TGA is advising health professionals and consumers that use of the diabetes medicine, pioglitazone, for more than a year may be associated with an increased risk of bladder cancer’ and noted that men were at substantially higher risk of bladder cancer than women stating; ‘To put these results into context, one thing to consider is the ‘normal’ incidence of bladder cancer. Incidence of bladder cancer varies from group to group, depending on risk factors such as age, gender, cigarette smoking and occupational exposure to certain chemicals. The Australian Institute of Health and Welfare estimated there would be around 2500 new cases of bladder cancer in Australia for 2010, about three‐quarters of these in males’.^4^ Age was listed as a risk factor for bladder cancer but no specific age groups were highlighted as being at greater risk.^4^ In contrast, the UK advisory did not mention patient sex as a risk for bladder cancer but stated under the new recommendations section that ‘Use in elderly patients should be considered carefully before and during treatment because the risk of bladder cancer increases with age’.^5^ Neither country provided direct advice to avoid use, but suggested that decisions be considered carefully in higher risks groups (see references for links to full advisory). It is not known whether the wording of these two advisories had different impacts on the use of pioglitazone in the specified age and sex groups.

The aim of this analysis was to compare the use of pioglitazone in Australia and the United Kingdom before and after the safety advisories were issued; and whether the impacts varied by sex and age group.

## 2 METHODS

### 2.1 Study period

The study period was defined as the period between 24‐months prior and the July 2011 safety advisories and the 11 months following the advisories (July 2009–June 2012), with July 2011 treated as a transition month.

### 2.2 Australian data

We analysed an extract of Pharmaceutical Benefits Scheme (PBS) data for a 10% random sample of beneficiaries (*N* = 1.4 million with claims for prescriptions during the time period under study). The PBS dataset captures dispensing of subsidised prescription medicines and covers all Australian citizens and permanent residents. The data extract included the generic name of the dispensed medicine, quantity supplied, date of dispensing, and the beneficiary's sex and year of birth. Australian population data for the entire resident population as well as by age and sex were obtained from the Australian Bureau of Statistics.^6^

### 2.3 UK data

We used data from the Clinical Practice Research Datalink (CPRD) Gold database for analysis of UK pioglitazone use. The dataset includes more than 11.3 million individuals from 674 general practices in England, Northern Ireland, Scotland and Wales, representing 6.9% of the British population, and is representative of the British population in terms of geography, socioeconomics, age and gender.^7^ CPRD Gold captures details of prescriptions written, as well as detailed demographic and clinical information.^7^

### 2.4 Outcome

The outcome measure for the study was the monthly rate of pioglitazone use, defined as the number of prescriptions dispensed (Australia) or written (UK) in that month per 100 000 population. Sex‐ and age‐based population estimates were used to calculate use per 100 000 population for sex and age subgroups.

### 2.5 Analysis

We carried out interrupted time series analyses to examine the monthly rate of pioglitazone use before and after the July 2011 safety advisories. Interrupted time series analysis was conducted for all individuals, by sex, and by age group (categorized as ‘under 65 years’ and ‘65 years and over’). The terms included in the model were: (i) the baseline monthly change in use prior to the safety advisories, July 2009–June 2011, (ii) the change in use occurring in the month after July 2011 (July was treated as a transitional month and not included in the models^9^), (iii) the monthly change in use for the 11 months after the safety advisories, August 2011–June 2012, and (iv) dummy variables for each month of the year to account for seasonality.

To account for autocorrelation, we used the SAS AUTOREG procedure to undertake regression modelling for each group.^8^ In each model generalized Durbin–Watson tests were used to detect autocorrelation. Where autocorrelation was found, we used stepwise autoregression to determine the order of autocorrelation.^8^ Autoregressive terms were fitted using Yule–Walker estimates. The total *R*‐square value was calculated to determine model goodness of fit. Results from the models were expressed as the estimated number of prescriptions per 100 000 population, standard error (SE) and *p*‐value. The regression co‐efficient for the baseline trend was extrapolated beyond June 2011 (to June 2012) to calculate the use that would have been expected had the safety advisories not been issued. For each series, we calculated the mean percentage change from the extrapolated baseline trend to the observed trend for the period after the safety advisories, and used non‐parametric bootstrapping resampling with replacement with 5000 iterations to estimate percentile‐based 95% confidence intervals (CIs).^10^, ^11^ The percentage change was compared for males and females, then for the two age groups using the test for interaction outlined by Altman and Bland.^12^ We calculated the difference between the percentage change in each group (e.g. males vs. females) and the 95% CIs for the difference, allowing us to determine whether the results for males and females, and for the two age groups were significantly different.^12^ The analytic code is available on request to the authors.

## 3 RESULTS

### 3.1 Overall use of pioglitazone

**FIGURE 1:**
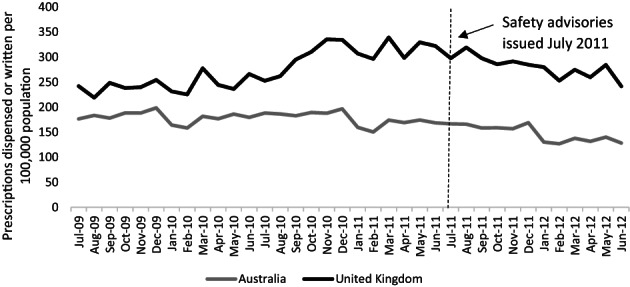
Number of pioglitazone prescriptions dispensed (Australia) or written (United Kingdom) per 100 000 population each month, July 2009 to June 2012. *Vertical broken line indicates the timing of the safety advisories, July 2011.

Figure 1 shows the overall use of pioglitazone in Australia and the United Kingdom for the study period (2 years before and 11 months after the July 2011 safety advisories). Use was higher in the United Kingdom than in Australia throughout the study period. In the month prior to the safety advisories, use was 322 prescriptions per 100 000 population in the United Kingdom, and 169 prescriptions per 100 000 population in Australia. Following the July 2011 safety advisories, overall use fell significantly in both countries; 17% in Australia (95% CI = −19.1 to −13.2) and 24% in the UK (95% CI = −28.1 to −19.8). In absolute terms, use fell to 128 prescriptions per 100 000 population by July 2012 in Australia and to 242 prescriptions per 100 000 population in the United Kingdom. Regression coefficients for all the regression models, including absolute changes, are provided in Table 1.

**TABLE 1:** Estimated number of pioglitazone prescriptions dispensed (Australia) or written (United Kingdom) per 100 000 population before and after the safety advisories in July 2011

		Australia				United Kingdom			
Group	Term	Total *R* ^2^	Prescriptions	SE	*p‐*Value	Total *R* ^2^	Prescriptions	SE	*p‐*Value
All	Prescriptions dispensed in July 2009	0.985	182.03	3.31	<0.001	0.949	203.29	16.92	<0.001
	Monthly change pre‐advisories		−0.178	0.21	0.421		5.22	0.67	<0.001
	Change in July 2011		−13.12	5.15	0.020		−37.30	25.29	0.179
	Monthly change post‐advisories		−2.53	0.66	0.001		−8.63	2.52	0.009
Males	Prescriptions dispensed in July 2009	0.989	226.58	4.14	<0.001	0.951	236.35	20.01	<0.001
	Monthly change pre‐advisories		−0.14	0.26	0.604		6.35	0.80	<0.001
	Change in July 2011		−18.28	6.48	0.023		−41.62	29.89	0.201
	Monthly change post‐advisories		−3.38	0.94	0.007		−9.99	2.97	0.001
Females	Prescriptions dispensed in July 2009	0.975	145.29	3.32	<0.001	0.946	170.81	13.79	<0.001
	Monthly change pre‐advisories		−0.13	0.21	0.551		4.12	0.54	<0.001
	Change in July 2011		−9.92	5.27	0.028		−32.81	20.60	0.150
	Monthly change post‐advisories		−2.12	0.67	0.002		−7.31	2.10	0.008
Age < 65 years	Prescriptions dispensed in July 2009	0.987	104.77	2.41	<0.001	0.953	115.19	8.52	<0.001
	Monthly change pre‐advisories		−0.41	0.15	0.0153		2.69	0.32	<0.001
	Change in July 2011		−8.84	3.66	0.026		−23.71	12.55	0.096
	Monthly change post‐advisories		−1.20	0.48	0.022		−5.04	1.31	0.005
Age ≥ 65	Prescriptions dispensed in July 2009	0.986	679.61	11.82	<0.001	0.951	616.76	53.40	<0.001
	Monthly change pre‐advisories		0.86	0.74	0.281		17.16	2.12	<0.001
	Change in July 2011		−54.02	19.70	0.025		−115.46	79.61	0.185
	Monthly change post‐advisories		−11.18	2.72	0.003		−26.50	7.92	0.010

### 3.2 Sex differences in use

**FIGURE 2:**
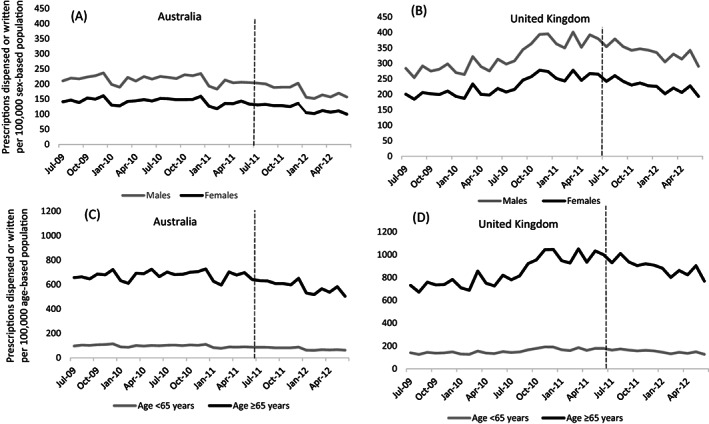
Number of pioglitazone prescriptions dispensed (Australia) or written (United Kingdom) per 100 000 population, July 2009 and June 2012, by sex (A,B) and age (C,D). Vertical broken lines indicate the timing of the safety advisories, July 2011

**FIGURE 3:**
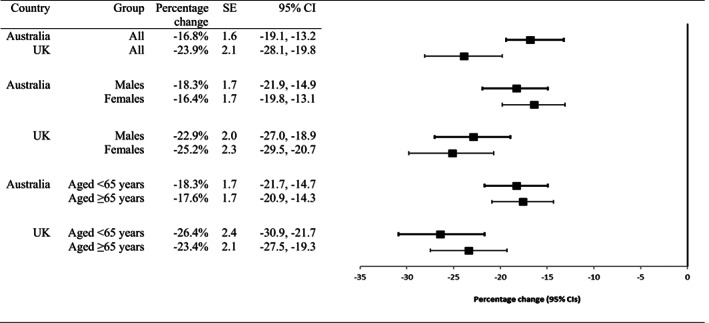
Percentage change (95% CIs) in prescriptions dispensed (Australia) or written (United Kingdom, UK) after the July 2011 safety advisories compared with the baseline trend, for all individuals and by sex and age

Figure 2 shows the use of pioglitazone by sex in Australia and the United Kingdom. Following the July 2011 safety advisories, pioglitazone use fell significantly for males and females in both countries (range −16% to −25%, Table 1). Use of pioglitazone was higher for males than females in Australia (Figure 2A) and the United Kingdom (Figure 2B) throughout the study period. In Australia, use by males fell by 18% in the 11 months following the safety advisory, and use by females fell by 16% (Figure 3). The difference between males and females was not statistically significant (difference − 1.9%, 95% CI = −6.6 to 2.8). In the United Kingdom, use by males and females fell by 23% and 25%, respectively, after the safety advisory. This difference was also non‐significant (difference 2.3%, 95% CI = −3.7 to 8.3).

### 3.3 Age differences in use

Following the July 2011 safety advisories pioglitazone use fell significantly for younger and older individuals in both countries (range −18% to −26%, Table 1). Pioglitazone use trends by age group for Australia and the UK are shown in Figure 2 (c and d). In both countries, use was considerably higher for people aged 65 years and over compared with younger people. Following the safety advisory in Australia, use of pioglitazone fell by 18% for both people aged 65 and over and those under 65 years (Figure 3). In the UK, use fell by 23% for people aged 65 and over and 26% for those under 65 years. The age‐group differences were not significant in either country (Australia 0.7%, 95% CI = ‐4.0 to −5.4; UK 3.0%, 95% CI = ‐3.3 to 9.3).

## 4 DISCUSSION

We examined sex‐ and age‐trends in pioglitazone use before and after safety advisories linking pioglitazone to bladder cancer were issued in Australia and the United Kingdom. The Australian advisory highlighted the higher background risk of bladder cancer in males whereas the UK advisory noted the increased risk in the elderly. We observed significant falls in pioglitazone use at population level for Australia (17%) and the United Kingdom (24%). Despite the larger relative decrease in pioglitazone use in the United Kingdom compared with Australia, absolute use remained higher in the United Kingdom at the end of the study period (242 vs. 128 prescriptions per 100 000 population in July 2012). Significant decreases in pioglitazone use were also seen for all sex and age subgroups in both countries following the July 2011 safety advisories (between 16%–18% in Australia, and 23%–26% in the United Kingdom), but the magnitude of the decreases did not differ by sex‐ or age‐group in either country. These findings indicate that the safety advisories reduced pioglitazone use overall in both countries, but that the highlighting of different at‐risk subgroups did not lead to varied reductions in use.

Our population‐level findings are consistent with those of two previous studies examining the impact of pioglitazone and bladder cancer safety advisories on pioglitazone use.^13^, ^14^, ^15^ A US study reported significant falls in pioglitazone prescriptions of 18% and 21%, respectively, in two regions following the US safety advisory (September 2010).^14^ In South Korea, the number of individuals dispensed pioglitazone fell by significantly 8% after the Korean safety advisory was issued (June 2011).^15^ Neither study compared the impact of the safety advisories on sex nor age.

By contrast, our population‐level findings differ from those reported in a previous Australian study.^13^ We reported a statistically significant reduction of 17% in Australia's use of pioglitazone following the July 2011 safety advisory. Another Australian study, using a similar analysis method and similar pre‐ and post‐intervention periods to our study reported a non‐significant decrease of 7% in pioglitazone use following the advisory.^13^ The discrepancy between this Australian study and our results is likely to be due to the data used. Our study used claims data based on date of supply to the patient, while the other Australian study used claims data based on date the pharmacy claim was processed by the Australian Government. Date of processing lags behind the date of supply, sometimes by months, and can lead to unreliable findings when specific time periods are examined.^16^ The discrepancy between date of supply and date of processing was particularly large in 2012 (the year following the safety advisory) due to changes in data reporting requirements for pharmacies.^17^

We did not observe age‐ or sex‐based differences in pioglitazone use following the July 2011 safety advisories in Australia or the United Kingdom. This lack of difference by sex or age may reflect a lack of direct advice in the text of both advisories. Although the UK advisory mentions the higher risk of bladder cancer in older people and that use should be ‘considered carefully before and during treatment’, there is no direct information provided on whether additional risks associated with pioglitazone use also increase with age.^5^ Additionally, the age‐related caution is not included within a section labelled ‘Advice for Health Professionals’, which directly states what prescribers should do.^5^ Similarly, the Australian advisory notes in the background section that the incidence of bladder cancer is higher in males, but a set of bullet points labelled ‘Information for Health Professionals’ makes no mention of avoiding use in men.^4^ In both cases, although regulators may have intended to warn against use in specific ‘at‐risk’ groups, this information was omitted from direct advice. A 10‐year comparison of safety advisories between regulators found large differences in the decision to warn,^18^ with little public access to information on the data or discussions underlying decision‐making.^19^ Evaluation of the risk communication strategies that contribute to intended changes in drug use have also been limited.^20^ Our current findings on patterns of pioglitazone use highlight the need for clear and prominent advice if the aim is to reduce use in at‐risk groups.

## 5 LIMITATIONS

We used interrupted time series analysis to compare pioglitazone use before and after the July 2011 safety advisories in Australia and the United Kingdom. Interrupted time series analysis is a powerful quasi‐experimental method which has been used widely to evaluate the impact of regulatory changes on medicines use.^21^, ^22^, ^23^, ^24^ However, we cannot rule out that falls in pioglitazone use we observed were due to factors other than the Australian and UK safety advisories. Trends from Australia were declining prior to the July 2011 safety advisories, while the trends in the United Kingdom appeared to be stabilizing in the 6 months prior to the advisory. This coincides with rapidly rising use of the gliptins (particularly sitagliptin) in Australia and the UK, the initial FDA warning about a possible link between pioglitazone and bladder cancer in September 2010, and the first published study warning of increased bladder cancer risk in pioglitazone users (April 2011).^1^, ^25^, ^26^, ^27^ It may be that clinicians were already favouring other clinical options for patients generally, and not just the subgroups identified in the Australian and UK advisories, prior to July 2011.

## 6 CONCLUSION

The results indicate that the specific information in the Australian and UK safety advisories highlighting at‐risk subgroups did not selectively influence pioglitazone use in the 11 months following the advisories. However, the general message communicating pioglitazone and bladder cancer risk was followed by substantial reductions in pioglitazone use at the population level in both countries.

## AUTHOR CONTRIBUTIONS

Barbara Mintzes, Elizabeth E. Roughead, Richard L. Morrow and Anna Kemp‐Casey conceived of the study. Anna Kemp‐Casey drafted the manuscript. Anna Kemp‐Casey performed the statistical analysis with assistance from Richard L. Morrow and Colin R. Dormuth. Elizabeth E. Roughead and Patrick C. Souverein assisted with acquisition of data. Elizabeth E. Roughead, Richard L. Morrow, and Barbara Mintzes interpreted the policy implications of the results and critically reviewed the manuscript. All authors read and approved the final manuscript.

## CONFLICT OF INTEREST

The authors have no conflicts to declare.

## ETHICS STATEMENT

This study was approved by the University of South Australia Human Research Ethics Committee, the Services Australia External Request Evaluation Committee, and the Independent Scientific Advisory Committee of the Medicines and Healthcare products Regulatory.
